# The genome sequence of the Buff-tailed Bumblebee, *Bombus terrestris* (Linnaeus, 1758)

**DOI:** 10.12688/wellcomeopenres.19248.1

**Published:** 2023-04-12

**Authors:** Liam M. Crowley, Olga Sivell, Duncan Sivell, University of Oxford and Wytham Woods Genome Acquisition Lab, Natural History Museum Genome Acquisition Lab, Darwin Tree of Life Barcoding collective, Wellcome Sanger Institute Tree of Life programme, Wellcome Sanger Institute Scientific Operations: DNA Pipelines collective, Tree of Life Core Informatics collective, Darwin Tree of Life Consortium

**Affiliations:** 1 University of Oxford, Oxford, England, UK; 2 Natural History Museum, London, England, UK

**Keywords:** Bombus terrestris, Buff-tailed Bumblebee, genome sequence, chromosomal, Hymenoptera

## Abstract

We present a genome assembly from an individual female *Bombus terrestris* (the Buff-tailed Bumblebee; Arthropoda; Insecta; Hymenoptera; Apidae). The genome sequence is 393.0 megabases in span. Most of the assembly is scaffolded into 18 chromosomal pseudomolecules. The mitochondrial genome has also been assembled and is 24.7 kilobases in length. Gene annotation of this assembly on Ensembl identified 14,435 protein coding genes.

## Species taxonomy

Eukaryota; Metazoa; Ecdysozoa; Arthropoda; Hexapoda; Insecta; Pterygota; Neoptera; Endopterygota; Hymenoptera; Apocrita; Aculeata; Apoidea; Apidae; *Bombus*; *Bombus*; *Bombus terrestris* (Linnaeus, 1758) (NCBI:txid30195).

## Background

The Buff-tailed Bumblebee, *Bombus terrestris*, is one of the seven most common and widespread species of bumblebees in the UK. It is generally common across its range that includes Europe, north Africa and the Near East ( Widmer *et al.*, 1998). It has become an established non-native species in Tasmania, Argentina, Japan and Chile ( Inoue *et al.*, 2008; Rendoll-Carcamo *et al.*, 2017; Semmens *et al.*, 1993; Torretta *et al.*, 2006). It is a eusocial species with reproductive queens and males, and non-reproductive workers. It is a large species with queens among the largest bumblebees in the UK reaching up to 22 mm in length. Workers exhibit a large degree of alloethism ( Goulson *et al.*, 2002). It is covered in black hairs, with bands of yellow hairs across the pronotum and second tergite, and white-to-buff hairs on the apical abdominal segments. It is part of the *Bombus lucorum* species aggregate, with workers being largely indistinguishable from those of *B. lucorum*, *B. magnus* and *B. cryptarum*. Queens and males can be distinguished from others in this group be their large size, deeper golden colour of the yellow hairs and buff, rather than white, colouration of the hairs on apical segments of the abdomen ( Edwards & Jenner, 2005). Larger workers occasionally have a narrow band of brownish hairs at the base of the white haired apical abdominal segments.

*Bombus terrestris* is broadly polylectic, visiting a wide range of flowers for pollen and nectar. It is not uncommon for this species to ‘nectar rob’ from flower species with deeper corollae, whereby a slit is cut in the base of the corolla to access the nectaries without entering the main flower. Nests are constructed underground, typically in old small-mammal burrows. Colonies are large and may reach up to around 500 workers at their peak. In UK agricultural landscapes, nest density is estimated at 29 nests per km ^2^, and minimum estimated maximum foraging range of 758 m ( Knight *et al.*, 2005). Typically, this species is univoltine with overwintered queens emerging from February to April, the first workers from March and males from July to October. In recent years, however, there have been increasing instances of winter active colonies of *B. terrestris* across southern UK cities. This may be in some part due to increasing winter temperatures and urban heat island effects, as well as the availability of winter blooming non-native flowers, such as Mahonia, in gardens.

In the UK wild populations are comprised of the subspecies *Bombus terrestris audax* ( Rasmont *et al.*, 2008). Due to its ability to be reared in captivity, this species has become a laboratory model species for bumblebees, and bees more generally. In particular, it has been extensively used in studies investigating the impacts of pesticides on pollinators (e.g. Gill & Raine, 2014) and insect pathology (e.g. Genersch *et al.*, 2006). Furthermore, it is commercially produced for crop pollination, especially in greenhouse systems where it is an effective pollinator of crops such as tomato ( Morandin *et al.*, 2001). The subspecies *Bombus terrestris dalmitinus* is imported to the UK for commercial pollination, leading to some concern regarding the conservation of the native subspecies following escape or release of imported non-natives ( Ings *et al.*, 2006).

The genome for this important laboratory model species will have myriad applications for investigations into areas such as the evolution of eusociality, conservation of important pollinator species, reproductive evolution and foraging behaviour. We note that a draft genome sequence for *B. terrestris* was generated previously ( Sadd *et al.*, 2015). Here we present a chromosomally complete genome sequence for *B. terrestris*, based on one female specimen from Wytham Woods, Oxfordshire, UK.

### Genome sequence report

The genome was sequenced from one female *Bombus terrestris* specimen collected from Wytham Woods, Oxfordshire, UK (latitude 51.77, longitude –1.34). A total of 57-fold coverage in Pacific Biosciences single-molecule HiFi long reads and 71-fold coverage in 10X Genomics read clouds were generated. Primary assembly contigs were scaffolded with chromosome conformation Hi-C data. Manual assembly curation corrected 33 missing joins or mis-joins and removed two haplotypic duplications, reducing the assembly length by 2.03% and the scaffold number by 4.96%, and increasing the scaffold N50 by 13.14%.

**Figure 1.**
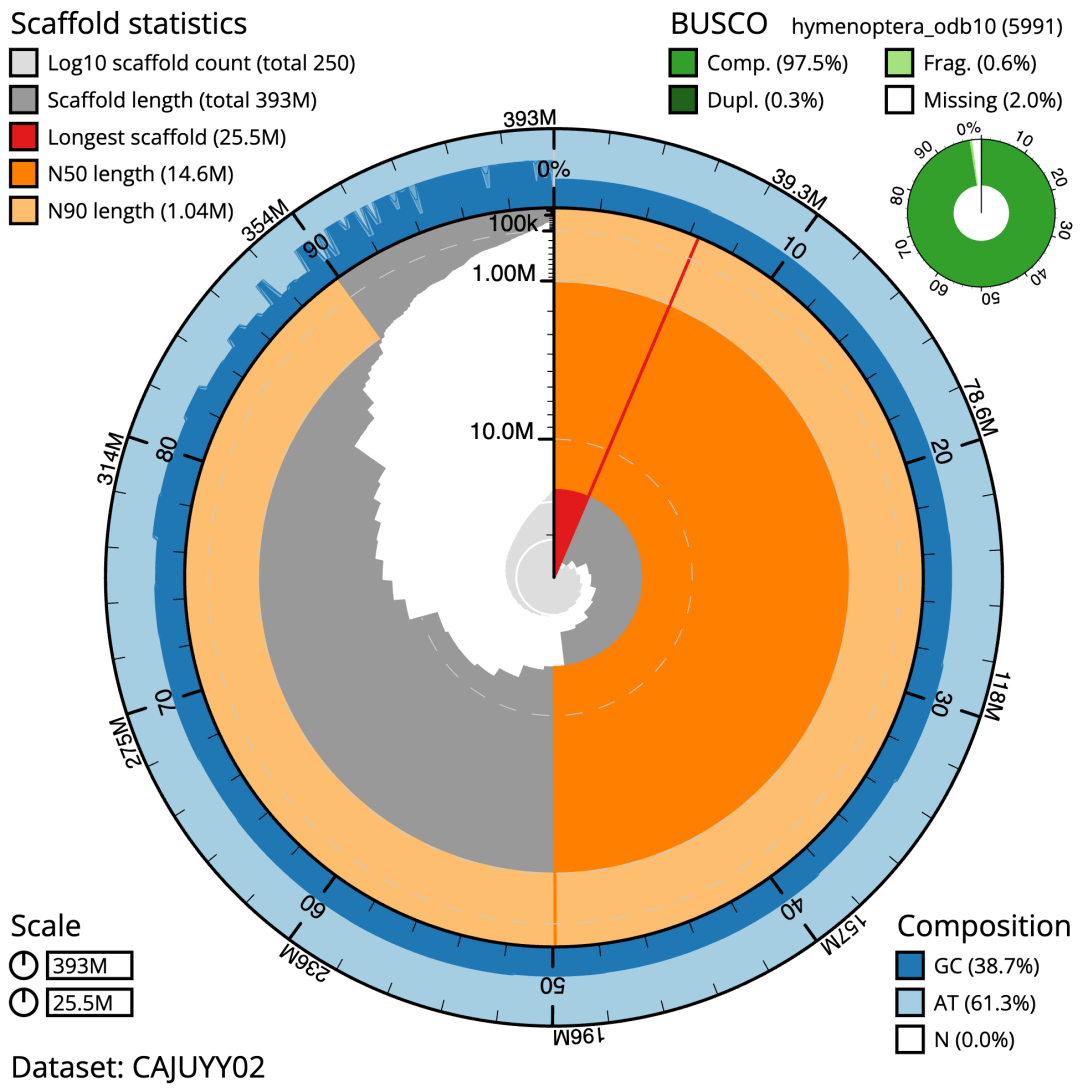
Genome assembly of *Bombus terrestris*, iyBomTerr1.2: metrics. The BlobToolKit Snailplot shows N50 metrics and BUSCO gene completeness. The main plot is divided into 1,000 size-ordered bins around the circumference with each bin representing 0.1% of the 392,986,749 bp assembly. The distribution of scaffold lengths is shown in dark grey with the plot radius scaled to the longest scaffold present in the assembly (25,524,254 bp, shown in red). Orange and pale-orange arcs show the N50 and N90 scaffold lengths (14,593,050 and 1,035,857 bp), respectively. The pale grey spiral shows the cumulative scaffold count on a log scale with white scale lines showing successive orders of magnitude. The blue and pale-blue area around the outside of the plot shows the distribution of GC, AT and N percentages in the same bins as the inner plot. A summary of complete, fragmented, duplicated and missing BUSCO genes in the hymenoptera_odb10 set is shown in the top right. An interactive version of this figure is available at https://blobtoolkit.genomehubs.org/view/iyBomTerr1.2/dataset/CAJUYY02/snail.

The final assembly has a total length of 393.0 Mb in 249 sequence scaffolds with a scaffold N50 of 14.6 Mb ( Table 1). Most (73.99%) of the assembly sequence was assigned to 18 chromosomal-level scaffolds. Chromosome-scale scaffolds are named by synteny based on the *B. terrestris* genome assembly GCA_000214255.1. Many unplaced scaffolds are composed entirely of a 10mer repeat which we note occurs in other Bombus assemblies ( Figure 1– Figure 4; Table 2). While not fully phased, the assembly deposited is of one haplotype. Contigs corresponding to the second haplotype have also been deposited. The estimated Quality Value (QV) of the final assembly is 50.1 with *k*-mer completeness of 99.96%, and the assembly has a BUSCO v5.3.2 ( Manni *et al.*, 2021) completeness of 97.5% (single 97.2%, duplicated 0.3%) using the hymenoptera_odb10 reference set ( *n* = 5,991).

**Table 1.** Genome data for *Bombus terrestris*, iyBomTerr1.2.

Project accession data		
Assembly identifier	iyBomTerr1.2	
Species	*Bombus terrestris*	
Specimen	iyBomTerr1	
NCBI taxonomy ID	30195	
BioProject	PRJEB45205	
BioSample ID	SAMEA7520487	
Isolate information	iyBomTerr1, female iyBomTerr2, female	
Assembly metrics *		*Benchmark*
Consensus quality (QV)	50.1	*≥ 50*
*k*-mer completeness	99.96%	*≥ 95%*
BUSCO **	C:97.5%[S:97.2%,D:0.3%], F:0.6%,M:2.0%,n:5,991	*C ≥ 95%*
Percentage of assembly mapped to chromosomes	73.99%	*≥ 95%*
Sex chromosomes	Not applicable	*localised homologous pairs*
Organelles	Mitochondrial genome assembled	*complete single alleles*
Raw data accessions		
PacificBiosciences SEQUEL II	ERR6558189	
10X Genomics Illumina	ERR6055006–ERR6055009	
Hi-C Illumina	ERR6055010, ERR6055011	
PolyA RNA-Seq Illumina	ERR6363272, ERR10123650	
Genome assembly		
Assembly accession	GCA_910591885.2	
*Accession of alternate haplotype*	GCA_910592195.2	
Span (Mb)	393.0	
Number of contigs	292	
Contig N50 length (Mb)	6.8	
Number of scaffolds	249	
Scaffold N50 length (Mb)	14.6	
Longest scaffold (Mb)	25.5	
Genome annotation		
Number of protein-coding genes	14,435	
Number of non-coding genes	5,285	
Number of gene transcripts	42,472	

* Assembly metric benchmarks are adapted from column VGP-2020 of “Table 1: Proposed standards and metrics for defining genome assembly quality” from ( Rhie *et al.*, 2021). ** BUSCO scores based on the hymenoptera_odb10 BUSCO set using v5.3.2. C = complete [S = single copy, D = duplicated], F = fragmented, M = missing, n = number of orthologues in comparison. A full set of BUSCO scores is available at https://blobtoolkit.genomehubs.org/view/iyBomTerr1.2/dataset/CAJUYY02/busco.

**Figure 2.**
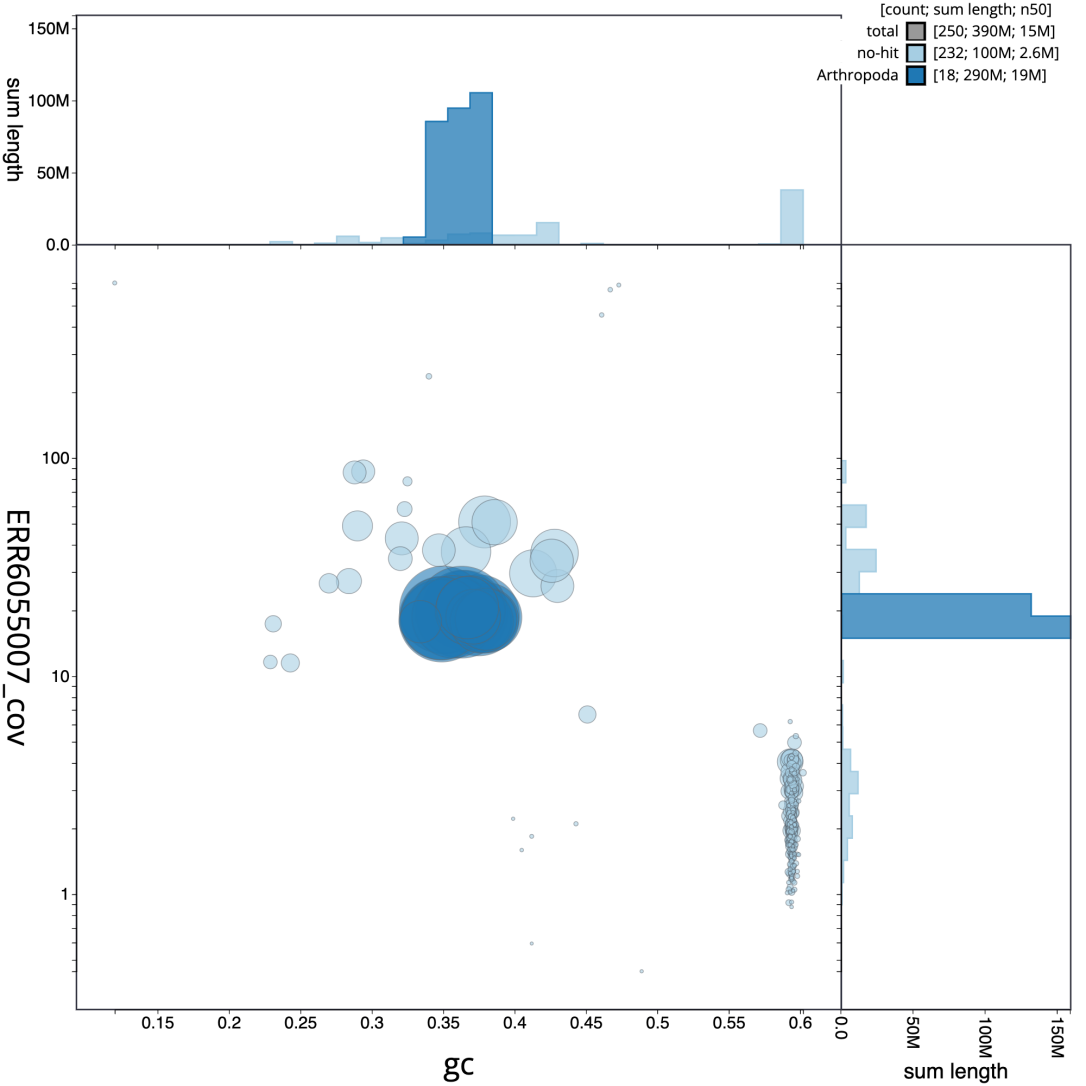
Genome assembly of *Bombus terrestris*, iyBomTerr1.2: GC coverage. BlobToolKit GC-coverage plot. Scaffolds are coloured by phylum. Circles are sized in proportion to scaffold length. Histograms show the distribution of scaffold length sum along each axis. An interactive version of this figure is available at https://blobtoolkit.genomehubs.org/view/iyBomTerr1.2/dataset/CAJUYY02/blob.

**Figure 3.**
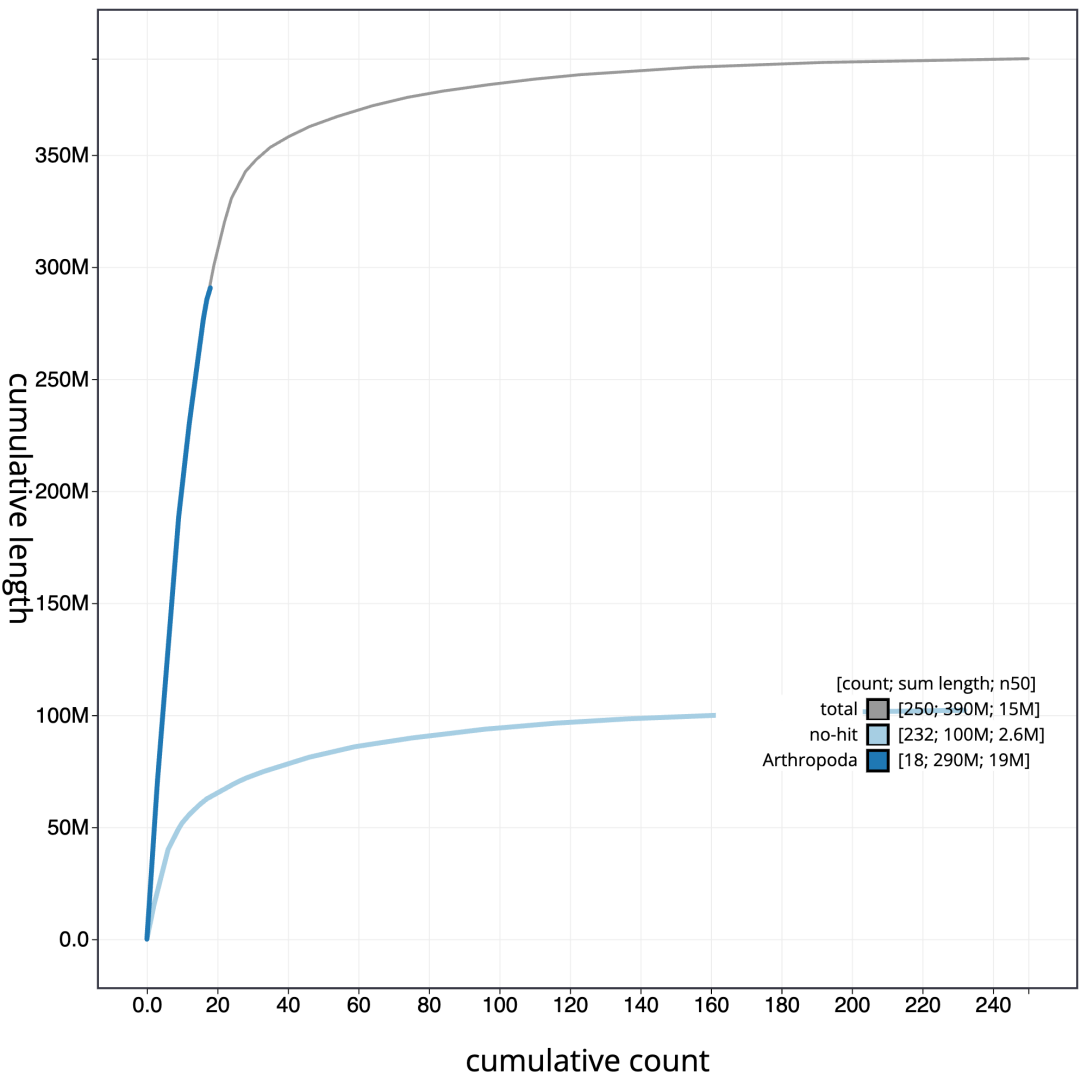
Genome assembly of *Bombus terrestris*, iyBomTerr1.2: cumulative sequence. BlobToolKit cumulative sequence plot. The grey line shows cumulative length for all scaffolds. Coloured lines show cumulative lengths of scaffolds assigned to each phylum using the buscogenes taxrule. An interactive version of this figure is available at https://blobtoolkit.genomehubs.org/view/iyBomTerr1.2/dataset/CAJUYY02/cumulative.

**Figure 4.**
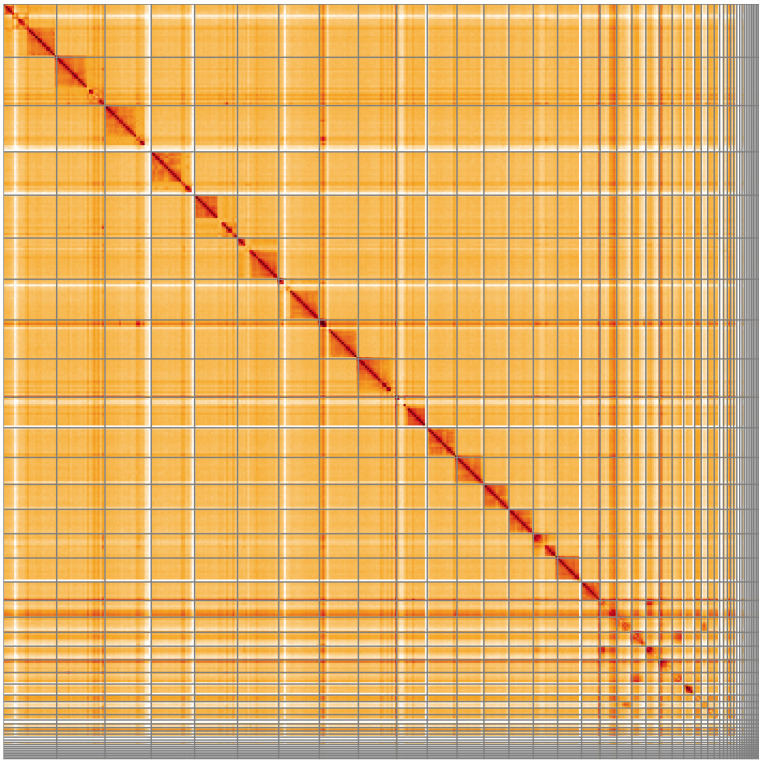
Genome assembly of *Bombus terrestris*, iyBomTerr1.2: Hi-C contact map. Hi-C contact map of the iyBomTerr1.2 assembly, visualised using HiGlass. Chromosomes are shown in order of size from left to right and top to bottom. An interactive version of this figure may be viewed at https://genome-note-higlass.tol.sanger.ac.uk/l/?d=ZQM9C3stRCyoai0oc0YK3w.

**Table 2.** Chromosomal pseudomolecules in the genome assembly of *Bombus terrestris*, iyBomTerr1.

INSDC accession	Chromosome	Size (Mb)	GC%
OU342921.1	1	18.37	36.3
OU342922.1	2	19.74	37.5
OU342923.1	3	22.1	34.9
OU342924.1	4	18.67	36.5
OU342925.1	5	14.19	38.1
OU342926.1	6	23.13	35
OU342927.1	7	25.52	36.3
OU342928.1	8	11.66	37.6
OU342929.1	9	19.45	34.8
OU342930.1	10	20.49	35.7
OU342931.1	11	20.78	34.9
OU342932.1	12	12.9	38
OU342933.1	13	14.59	36.9
OU342934.1	14	11.96	37.7
OU342935.1	15	11.41	38
OU342936.1	16	8.89	37.1
OU342937.1	17	11.65	36.7
OU342938.1	18	5.27	33.4
OU342939.1	MT	0.02	12.1
-	unplaced	102.19	45.5

### Genome annotation report

The *Bombus terrestris* genome assembly GCA_910591885.2 (iyBomTerr1.2) was annotated using the Ensembl rapid annotation pipeline ( Table 1; https://rapid.ensembl.org/Bombus_terrestris_GCA_910591885.2/Info/Index/). The resulting annotation includes 42,472 transcribed mRNAs from 14,435 protein-coding and 5,285 non-coding genes.

## Methods

### Sample acquisition and nucleic acid extraction

A female *Bombus terrestris* (iyBomTerr1) was collected from Wytham Woods, Oxfordshire (biological vice-county Berkshire), UK (latitude 51.77, longitude –1.34) on 7 August 2019. The specimen was taken from woodland habitat by Liam Crowley (University of Oxford) by netting. The specimen was identified by the collector and snap-frozen on dry ice.

A second female *B. terrestris* specimen (iyBomTerr2) was used for RNA sequencing. The iyBomTerr2 specimen was collected by Olga Sivell (Natural History Museum) from woodland edge in Luton, UK (latitude 51.88, longitude –0.37) on 6 May 2020. The specimen was identified by Duncan Sivell (Natural History Museum) and snap-frozen on dry ice.

DNA was extracted at the Tree of Life laboratory, Wellcome Sanger Institute (WSI). The iyBomTerr1 sample was weighed and dissected on dry ice with tissue set aside for Hi-C sequencing. Head and thorax tissue was cryogenically disrupted to a fine powder using a Covaris cryoPREP Automated Dry Pulveriser, receiving multiple impacts. High molecular weight (HMW) DNA was extracted using the Qiagen MagAttract HMW DNA extraction kit. Low molecular weight DNA was removed from a 20 ng aliquot of extracted DNA using the 0.8X AMpure XP purification kit prior to 10X Chromium sequencing; a minimum of 50 ng DNA was submitted for 10X sequencing. HMW DNA was sheared into an average fragment size of 12–20 kb in a Megaruptor 3 system with speed setting 30. Sheared DNA was purified by solid-phase reversible immobilisation using AMPure PB beads with a 1.8X ratio of beads to sample to remove the shorter fragments and concentrate the DNA sample. The concentration of the sheared and purified DNA was assessed using a Nanodrop spectrophotometer and Qubit Fluorometer and Qubit dsDNA High Sensitivity Assay kit. Fragment size distribution was evaluated by running the sample on the FemtoPulse system.

RNA was extracted from thorax tissue of iyBomTerr2 in the Tree of Life Laboratory at the WSI using TRIzol, according to the manufacturer’s instructions. RNA was then eluted in 50 μl RNAse-free water and its concentration assessed using a Nanodrop spectrophotometer and Qubit Fluorometer using the Qubit RNA Broad-Range (BR) Assay kit. Analysis of the integrity of the RNA was done using Agilent RNA 6000 Pico Kit and Eukaryotic Total RNA assay.

### Sequencing

Pacific Biosciences HiFi circular consensus and 10X Genomics read cloud DNA sequencing libraries were constructed according to the manufacturers’ instructions. Poly(A) RNA-Seq libraries were constructed using the NEB Ultra II RNA Library Prep kit. DNA: and RNA sequencing were performed by the Scientific Operations core at the WSI on Pacific Biosciences SEQUEL II (HiFi), Illumina HiSeq 4000 (RNA-Seq) and HiSeq X Ten (10X) instruments. Hi-C data were also generated from tissue of iyBomTerr1 using the Arima v2 kit and sequenced on the HiSeq X Ten instrument.

### Genome assembly, curation and evaluation

Assembly was carried out with Hifiasm ( Cheng *et al.*, 2021) and haplotypic duplication was identified and removed with purge_dups ( Guan *et al.*, 2020). One round of polishing was performed by aligning 10X Genomics read data to the assembly with Long Ranger ALIGN, calling variants with FreeBayes ( Garrison & Marth, 2012). The assembly was then scaffolded with Hi-C data ( Rao *et al.*, 2014) using SALSA2 ( Ghurye *et al.*, 2019). The assembly was checked for contamination and corrected using the gEVAL system ( Chow *et al.*, 2016) as described previously ( Howe *et al.*, 2021). Manual curation was performed using gEVAL, HiGlass ( Kerpedjiev *et al.*, 2018) and Pretext ( Harry, 2022). The mitochondrial genome was assembled using MitoHiFi ( Uliano-Silva *et al.*, 2022), which performed annotation using MitoFinder ( Allio *et al.*, 2020). To evaluate the assembly, MerquryFK was used to estimate consensus quality (QV) scores and *k*-mer completeness ( Rhie *et al.*, 2020). The genome was analysed and BUSCO scores ( Manni *et al.*, 2021; Simão *et al.*, 2015) were generated within the BlobToolKit environment ( Challis *et al.*, 2020). Table 3 contains a list of software tool versions and sources.

**Table 3.** Software tools: versions and sources.

Software tool	Version	Source
BlobToolKit	4.0.7	https://github.com/blobtoolkit/ blobtoolkit
BUSCO	5.3.2	https://gitlab.com/ezlab/busco
FreeBayes	1.3.1-17- gaa2ace8	https://github.com/freebayes/ freebayes
gEVAL	N/A	https://geval.org.uk/
Hifiasm	0.12	https://github.com/chhylp123/ hifiasm
HiGlass	1.11.6	https://github.com/higlass/higlass
Long Ranger ALIGN	2.2.2	https://support.10xgenomics.com/ genome-exome/software/pipelines/ latest/advanced/other-pipelines
Merqury	MerquryFK	https://github.com/thegenemyers/ MERQURY.FK
MitoHiFi	2	https://github.com/marcelauliano/ MitoHiFi
PretextView	0.2	https://github.com/wtsi-hpag/ PretextView
purge_dups	1.2.3	https://github.com/dfguan/purge_ dups
SALSA	3	https://github.com/salsa-rs/salsa

### Genome annotation

The Ensembl gene annotation system ( Aken *et al.*, 2016) was used to generate annotation for the *Bombus terrestris* assembly (GCA_910591885.2). Annotation was created primarily through alignment of transcriptomic data to the genome, with gap filling via protein-to-genome alignments of a select set of proteins from UniProt ( UniProt Consortium, 2019).

### Ethics and compliance issues

The materials that have contributed to this genome note have been supplied by a Darwin Tree of Life Partner. The submission of materials by a Darwin Tree of Life Partner is subject to the Darwin Tree of Life Project Sampling Code of Practice. By agreeing with and signing up to the Sampling Code of Practice, the Darwin Tree of Life Partner agrees they will meet the legal and ethical requirements and standards set out within this document in respect of all samples acquired for, and supplied to, the Darwin Tree of Life Project. All efforts are undertaken to minimise the suffering of animals used for sequencing. Each transfer of samples is further undertaken according to a Research Collaboration Agreement or Material Transfer Agreement entered into by the Darwin Tree of Life Partner, Genome Research Limited (operating as the Wellcome Sanger Institute), and in some circumstances other Darwin Tree of Life collaborators.

## Data Availability

European Nucleotide Archive: *Bombus terrestris* (buff-tailed bumblebee). Accession number PRJEB45205; https://identifiers.org/ena.embl/PRJEB45205. ( Wellcome Sanger Institute, 2021) The genome sequence is released openly for reuse. The *Bombus terrestris* genome sequencing initiative is part of the Darwin Tree of Life (DToL) project. All raw sequence data and the assembly have been deposited in INSDC databases. Raw data and assembly accession identifiers are reported in Table 1.

## Author information

Members of the University of Oxford and Wytham Woods Genome Acquisition Lab are listed here: https://doi.org/10.5281/zenodo.4789928.

Members of the Natural History Museum Genome Acquisition Lab are listed here: https://doi.org/10.5281/zenodo.4790042.

Members of the Darwin Tree of Life Barcoding collective are listed here: https://doi.org/10.5281/zenodo.4893703.

Members of the Wellcome Sanger Institute Tree of Life programme are listed here: https://doi.org/10.5281/zenodo.4783585.

Members of Wellcome Sanger Institute Scientific Operations: DNA Pipelines collective are listed here: https://doi.org/10.5281/zenodo.4790455.

Members of the Tree of Life Core Informatics collective are listed here: https://doi.org/10.5281/zenodo.5013541.

Members of the Darwin Tree of Life Consortium are listed here: https://doi.org/10.5281/zenodo.4783558.
