# Supplementary material for: The genome sequence of the Buff-tailed Bumblebee, Bombus terrestris (Linnaeus, 1758)
Source: Wellcome Open Res. 2023 Apr 12;8:161. [Version 1] doi: 10.12688/wellcomeopenres.19248.1 (PMC10818103; doi:10.12688/wellcomeopenres.19248.1)
Supplement: Peer review history

# Reviewer report: Reviewer response for version 1

**DOI:** 10.21956/wellcomeopenres.21331.r70845

**Published:** 2024-01-26

**Authors:** Natalia Araujo

**Affiliations:** 1 Unit of Evolutionary Biology & Ecology –, Universite Libre de Bruxelles, Brussels, Brussels, Belgium

The article reports an updated genome assembly for the Buff-tailed bumblebee Bombus terrestris based on the DNA of a single female. The assembly and analyses pipeline is the standard and well-established pipeline used by the Darwin Tree of Life project. There is no major reason for rejecting this manuscript report. Still, a few points could be improved as explained below.

 Both nuclear and mitochondrial genomes reported are larger than the previous assemblies for the species. Notably, the nuclear genome has had an increase of over 100Mb of genomic data. This seems like a good addition to the community. Interestingly though, about 100Mb of data was unplaced in chromossomes. So, this increase could be explained by the 10mer repeats of high GC content observed, which is likely composed of repetitive DNA and could be variable across sampled specimens. Maybe this should be mentioned more clearly in the manuscript body, or even better, a brief comparison between the previous assembly and the new one reported should be added so the community can better evaluate how this new assembly is an improvement from the old one and how it could impact future genomic analyses.

 I also believe the report could be enhanced by including a results section about the mitochondrial genome assembly, as the total size obtained 24.7kb greatly deviates from the standard bee mitochondrial genome size (~17kb). Is this only due to duplications? Has it increased repetitive content?

Are sufficient details of methods and materials provided to allow replication by others?

Yes

Is the rationale for creating the dataset(s) clearly described?

Yes

Are the datasets clearly presented in a useable and accessible format?

Yes

Are the protocols appropriate and is the work technically sound?

Yes

Reviewer Expertise:

Bee genomics

I confirm that I have read this submission and believe that I have an appropriate level of expertise to confirm that it is of an acceptable scientific standard.

# Reviewer report: Reviewer response for version 1

**DOI:** 10.21956/wellcomeopenres.21331.r70846

**Published:** 2023-12-27

**Authors:** Yannick Wurm

**Affiliations:** 1 Bioinformatics, Queen Mary University of London, London, England, UK

The authors report a new genome assembly for Bombus terrestris. 

 1. A previous high-quality genome for this species was published in 2015. That project involved substantial manual curation, including through the use of genetic maps and human specialist gene curators. It is also chromosome-level. It is thus surprising to see no comparison to the quality of the pre-existing standard, as that makes it unclear whether this new genome is an improvement or a step backwards. Can the authors provide comparative metrics for the two assemblies? And for the derived genesets? 

 2. The previous genome can also be helpful for some of the superscaffolding that may still want to take place here. 

 3. The authors cite some relevant naturalist background information for this species. It would also be relevant to highlight how it is *the* main model for genomic studies in bumblebees, including for understanding the genetics of its environmental adaptation Thomas J Colgan et al. (2022 ^1^) . 

 4. This species is also commonly called the "large earth bumblebee". That name should be mentioned. 

 5. The 10mer repeat is interesting. What is it? 

 6. Table 2 refers to a different version of the genome than figure 4?

Are sufficient details of methods and materials provided to allow replication by others?

Yes

Is the rationale for creating the dataset(s) clearly described?

No

Are the datasets clearly presented in a useable and accessible format?

Yes

Are the protocols appropriate and is the work technically sound?

Yes

Reviewer Expertise:

bee health and genomics

I confirm that I have read this submission and believe that I have an appropriate level of expertise to confirm that it is of an acceptable scientific standard, however I have significant reservations, as outlined above.

## References

[1] : Genomic Signatures of Recent Adaptation in a Wild Bumblebee. *Mol Biol Evol* .2022;39(2) : 10.1093/molbev/msab366 10.1093/molbev/msab366 35134226 PMC8845123

# Reviewer report: Reviewer response for version 1

**DOI:** 10.21956/wellcomeopenres.21331.r70837

**Published:** 2023-12-23

**Authors:** Pilar De la Rúa

**Affiliations:** 1 Department of Zoology and Physical Anthropology, University of Murcia, Murcia, Spain

This manuscript presents the details of the process of obtaining the sequencegenome assembly of the important pollinator Bombus terrestris.

 The work is straightforward and the whole process is well detailed. I have only a few editing notes to add:

 - dalmitinus should be changed to dalmatinus

 - this article should be mentioned:

 Hart et al. (2021 ^1^)

 - what´s the difference between this and the previous draft genome sequence? a short sentence justifying the need for this new genome sequence would be desirable

Are sufficient details of methods and materials provided to allow replication by others?

Yes

Is the rationale for creating the dataset(s) clearly described?

Yes

Are the datasets clearly presented in a useable and accessible format?

Yes

Are the protocols appropriate and is the work technically sound?

Yes

Reviewer Expertise:

pollinators conservation genetics

I confirm that I have read this submission and believe that I have an appropriate level of expertise to confirm that it is of an acceptable scientific standard.

## References

[1] : Winter activity unrelated to introgression in British bumblebee Bombus terrestris audax. *Apidologie* .2021;52(2) : 10.1007/s13592-020-00822-w 315-327 10.1007/s13592-020-00822-w
